# Outcome of burn injury and associated factor among patient visited at Addis Ababa burn, emergency and trauma hospital: a two years hospital-based cross-sectional study

**DOI:** 10.1186/s12873-022-00758-7

**Published:** 2022-12-09

**Authors:** Damena Mulatu, Ayalew Zewdie, Biruktawit Zemede, Bewuketu Terefe, Bikis Liyew

**Affiliations:** 1 Department of internal medicine, School of medicine, College of Medicine and Health Sciences, University of Gondar, Gondar, Ethiopia; 2 Department of Emergency Medicine and Critical Care, St Paul’s Hospital Millennium Medical College, Addis Ababa, Ethiopia; 3 Department of Community Health Nursing, School of Nursing, College of Medicine and Health Sciences, University of Gondar, Gondar, Ethiopia; 4 Department of Emergency and Critical Care Nursing, School of Nursing, College of Medicine and Health Sciences, University of Gondar, P.O.BOX 196, Gondar, Ethiopia

**Keywords:** Burn injuries, Magnitude, Outcome, Total body surface area percentage, Addis Ababa burn, Emergency and trauma hospital

## Abstract

**Background:**

Burn is one of the critical health problems worldwide. Developing countries with sub-Saharan and Asian populations are affected more. Its mortality and non-fatal complications depend on several factors including age, sex, residency, cause, the extent of the burn, and time and level of care given.

**Objective:**

The purpose of this study was to assess the outcome of burn injury and its associated factor among patients who visited Addis Ababa burn emergency and trauma hospital.

**Methods:**

The institutional-based, retrospective cross-sectional study design was conducted from April 1, 2019, to March 30, 2021. After checking the data for its consistency the data were entered and analyzed by using SPSS version 25. A total of 241 patients who had visited Addis Ababa burn, emergency and trauma Hospitals after sustained burn injury were recruited through convenience sampling method for final analysis. Model goodness-of-fit was checked by Hosmer and Lemeshow test (0.272). After checking multi-collinearity both the bi-variable and multivariable logistic regression model was fitted and variables having a *p*-value less than or equal to 0.05 at 95% CI in the multivariable analysis were considered statistically significant.

**Result:**

Adults (age 15 to 60 years) are the most affected groups accounting for 55.2% followed by pediatric age groups (age <15 years) (43.6%) and the elderly (age > 60 years) (1.2%). Scald burn was the major cause accounting for 39 % followed by Flame burn (33.6%), Electrical burn (26.6%), and chemical burn (0.8%). The mean TBSA% was 15.49%, ranging from1% to 64%. Adult males are more affected by electrical burns while adult females and the elderly encounter flame burn. 78.4% of patients were discharged without complications, 14.9% were discharged with complications and 6.6% died. The commonest long-term complication is the amputation of the extremity (19, 7.9%). Age greater than 60 years and TBSA% greater than 30% is a strong predictors of mortality with odds of 2.2 at 95% CI of [1.32, 3.69] and 8.7 at 95% CI of [1.33, 57.32] respectively.

**Conclusion and recommendation:**

The mortality rate show decrement from previous studies. Overall scald burn is common in all age groups but electrical burns and flame burns affected more adult and elderly age groups. Extremities were by far, the commonest affected body parts. The extent of burn injury and the age of the patient independently predict mortality. Early intervention will reduce mortality and complications.

## Background

Human skin and other underlying organs can be destroyed or damaged by burns [1] and categorized according to extent of damage to the body part [1]. Timely adequate resuscitation decrease both mortality and nonfatal complications associated with burn injury [1–3]. According to a WHO report of 2018, over 200-300,000 people die from fire-related burns annually, disproportionately in low and middle-income countries of African and Asian countries [4–6]. 90% of burn occurs in low and middle-income countries [7]. And also WHO 2018 report shows that around 265,000 burn-related deaths occur annually. Most of them occur in low and middle-income African and Asian countries accounting for more than 90% of death [8–11]. Death by burn injury in low- and middle-income countries (LMICs) is estimated to be eleven times higher than in high-income countries as the World Health Organization (WHO) estimated that 43,000 people die of burns in Africa every year with a rate of 6.1 per 100,000 [12]. In the USA, one million people seek medical care for burns annually, with an estimated mortality rate of 3 – 5% [13]. Most burns occur at home, causes being due to scald and flame [14]. Different studies reported that children are most at risk for burn injuries [15–21]. Most studies highlight younger males and older females as most at risk for burn injuries [15–19, 22], although some showed no clear differences between the sexes [21, 23]. Scald and contact injuries appear to be the most common [15–17, 22, 23], often as a result of bathing or cooking, however, flame injuries, as well as electrical and chemical burns, are common in some areas [21]. In the studies describing the location of the burn injuries, the extremities – particularly the upper extremities – are the most common locations for suffering burn injuries, especially in scald and contact burns. The lower extremities are more commonly affected by flame injuries [17, 21, 23]. Many studies highlight the home, particularly the kitchen, as the most common location for sustaining burn injuries [15–18, 22]. Children under the age of five who sustained burn injuries were 69% and over 70% of burns are sustained at home [24, 25]. Even if the trend of burning is decreasing in most developed nations, the problem is still on a rise in most developing countries including Ethiopia [4, 8, 26]. Mortality and morbidity are burdened more in developing countries [8–10, 26]. Each year approximately about 11 million peoples suffer from burns making almost 30,000 new burn injuries per day with an annual mortality rate of around 200,000 to 300,000 per year [8, 13]. The rate of burn-related deaths has been declining in many high-income nations, whereas it is currently nearly 7 times greater in low- and middle-income countries than in high-income ones for children [4, 13]. Non-fatal burn injuries are also a leading cause of morbidity including prolonged hospitalization, disfigurement, and disability often resulting in stigma and rejection [4, 11, 27]. In Europe, the in-hospital crude mortality ranges from 1.4% to 18% and is mostly influenced by regional economic conditions and variations in the case mix (TBSA percent) [2]. America, Eastern Medetrinian, and the Western Pacific regions, which are grouped as underdeveloped countries, account for 1.3, 0.02, and 0.6%, not even 2% combined of global mortality due to fires related death, respectively [8]. A study from a major burn center in Southwest China shows improvement from time to time as early intervention and prevention were applied. The mortality rate from the research result was 0.9 % [26]. In sub-Saharan African countries the severity and mortality rate is different in a different country with an average mortality rate of 17%, a death of one for every five burn victim [5]. In some African countries, the Mortality rate exceeds the average one. E.g. Togo 24.6% [28], Malawi 27% [29].

The outcome of the burn depends on the age and sex of the patient, burn surface area, depth of burn, burn site, type of burn, time of arrival to the hospital, getting appropriate resuscitation, presence of comorbidities as well as presence of rehabilitative center with the trained practitioner [11, 26, 30–32]. Inhalational injury is an independent risk factor for death [31, 33]. The commonest age of burn injury is 5 -14 years with the highest mortality rate in children less than 5 years and elderly greater than 70 years [10]. A study done in Taiwan reported that those aged over 65 years constitute 13- 20% of admissions to burn units n [34]. Females in South-East Asia have the highest fire-related burn mortality rates worldwide, followed by males in Africa and females in the Eastern Mediterranean [10, 34]. Burns greater than 15% in an adult and greater than 10% in a child have a poor prognosis [4, 11]. The mortality rate is higher for those that have higher TBSA%, third rather than second-degree burn, and inhalational injury [8, 34, 35]. Developing nations show low engagement rate than developed countries, probably because other competing diseases need to be controlled [8]. Advances in critical care (fluid resuscitation, nutritional assistance, and antibiotic treatment), as well as the adoption of early burn wound excision, play a substantial role in this improved survival rate [2]. Mortality and non-fatal injuries depend on several factors including age, sex, place of residency, economic status, involved site, depth, TBSA % involved, co-morbidity, time arrival to the emergency department, pre-hospital care, and presence of trained staff in giving the appropriate level of care [1, 4, 34]. A community-based study in Northern Ethiopia showed that the most commonly affected age group were children less than 10 years of age, and scald burn and domestic settings are the predominant causes and settings responsible for burns in the pediatric population, respectively [36, 37]. Different studies showed that the majority of burn patients were discharged without complication [14, 36, 38, 39]. In Ethiopia burn accounts for 1.5% to 9% of injuries in all age groups and between 4% and 15% of injuries in the pediatric population with an estimated mortality rate of 11.6% [8, 40]. The mortality rate according to a study conducted in Addis Ababa, Ethiopia was 11.6% [41]. In a rural community survey in Ethiopia, burns were the second most common injury to children under 15 years of age [13]. Research done at Bahir-dar, Ethiopia showed that 0.46% of pediatric female populations were more affected than males [42]. In Ethiopia, there is little research conducted related to burn injury and they focused on pattern and causative factors mainly, not the outcome of burn injury. The available information’s also somewhat incomplete [43]. After burn injuries were raised as a major health problem in the country, AaBET hospital was established to give care to burn and trauma victims 6 years ago. Since then, the hospital continued to give care to such victims. Since its establishment however published data on the outcome of burn injuries is not yet available. Therefore the significance of this study will be to assess the outcome of burn injury and its associated factor among burn patients who visited the hospital. This study is to analyze the outcome of burn injury and its associated factor among patients who visited AaBET Hospital from April 1, 2019, to march 30, 202.

## Conceptual framework

**Fig. 1:**
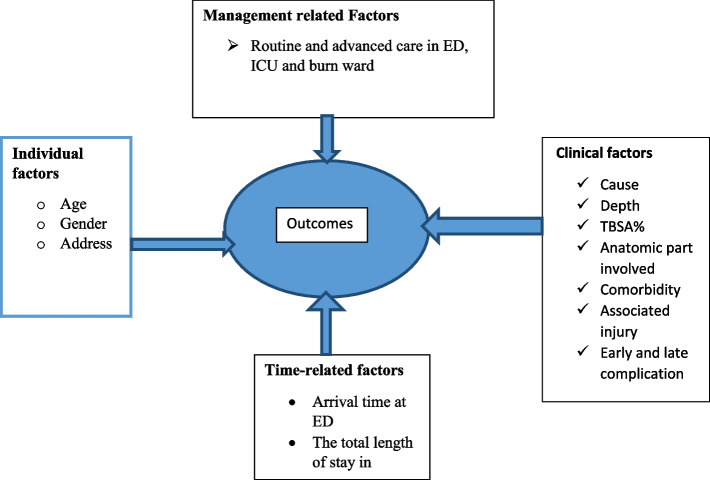
Conceptual framework showing the outcome of burn injury and associated factors

The outcome of burn injury is affected by different factors. These factors are divided into four groups. The Conceptual framework shown in the figure below helps to summarize the relationship between outcomes of burn injury with its associated factor (Fig. 1).

## Methodology

### Study design and period

A hospital-based cross-sectional study was conducted from May 1, 2021, to October 31, 2021.

### Study Setting

This study was conducted in Addis Ababa Burn, Emergency, and Trauma (AaBET) hospital, located in Addis Ababa, Ethiopia. AaBET hospital had a large Emergency unit labeled as a red to green area, two general ICU care setups with 11 functional beds, 3 semi-ICU beds, and a separate burn unit. All burn patients 1^st^ visit emergency units before admission to the burn unit for completing initial resuscitation. The emergency room has 5 resuscitation beds on the red side, 12 beds on the orange, and > 12 beds in a yellow-green area in which, any patient can be treated. It also has a separate decontamination room. Emergency and ICU setup is run by the EMCC department. Interns, residents, and nursing staff under the supervision of EMCC consultants manage the patient in collaboration with other department staff. The care will be according to the patient's needs, from routine care to advanced care. The burn unit is under the Plastic and reconstructive surgery department with 19 functional beds (7 pediatric and 12 adults). There are 32 nurses specially trained in burn care, 6 plastic surgeons, 5 residents, 6 General practitioners, and one dedicated operating room.

### Population

#### Source population

All patients visited Addis Ababa Burn, Emergency, and Trauma (AaBET) hospital from April 1, 2019, to March 31, 2021.

#### Study Population

All burn patients who visited Addis Ababa Burn, Emergency and Trauma (AaBET) hospital from April 1, 2019, to March 31, 2021, meet the inclusion criteria included in this study.

### Eligibility criteria

#### Inclusion criteria

- ✓ All burn patients who visited Addis Ababa Burn, Emergency, and Trauma (AaBET) hospital from April 1, 2019, to March 31, 2021, and eligible for the study.

#### Exclusion criteria

- ✓ Those burned patients were dead bodies on arrival and not restituted in the emergency department.
- ✓ All inadequate or incomplete files such as the absence of age, sex, diagnosis, outcome, burn depth, burn surface area, management plan
- ✓ That patient left against medical advice or transferred to another hospital
- ✓ Those treated on an outpatient bases

### Study variables

The dependent variable was the outcome of the burn patient (death or survival). The independent variables included socio-demographic variables, triage category, total body surface area percentage(TBSA%) burnt, depth of burn injury at admission, length of stay, Cause of burn, arrival time to ED, a complication in Stay at the hospital, and co-morbidity.

### Sample size determination and sampling method

All burn patients who visited Addis Ababa Burn, Emergency and Trauma (AaBET) Hospital from April 1, 2019, to March 31, 2021, who fulfilled inclusion criteria during the study period were included. All eligible medical records were included to get a good conclusion by using the convenience sampling method.

### Data collection tools and procedures

A data abstraction tool was prepared by the principal investigator in English by reviewing different literature to explore the objectives of the study. The sources of data were secondary data from medical records of burn victims. A structured checklist was adopted from different literature [44, 45]. Data collectors were Bachelor of Science (BSc) nurses and they were trained on the variables. The collected data were reviewed daily by the principal investigator. The questionnaire contains demographic characteristics (age, gender & address), clinical factors (cause, depth, total body surface area percentage (TBSA%) burnt, involved body part, comorbidity & associated injury), management-related factors (pre-hospital interventions, care given in hospital, early and late complications encountered and cause of death) and time-related factors (time of arrival to hospital & length of stay in emergency departments, Burn ward and intensive care units (ICU). From two-year retrospective data, from April 01, 2019, to March 31, 2021, a total of 268 patients who had visited Addis Ababa Burn, Emergency, and Trauma Hospital after sustaining burn injury were found. Of that 10 charts are lost and 6 cards are incomplete and excluded from the analysis. A total of 252 patients had completed the chart. Among them, 8 of them went against clear documented medical advice, and 3 were transferred to another hospital. So these patients were also excluded due to the difficulty of the conclusion of patient status.

### Data quality control

All data are checked for clarity, completeness, and correct recording of all the necessary information by the principal investigator. The quality of data was further ascertained during the data entry and cleaning process**.** A structured and standardized questionnaire developed in the English language by the principal investigator was used. It was tested for validity on 10% of charts before the start of data collection by the principal investigator. Data were collected by trained data collectors. They were trained by the principal investigator on when, how, and how much data should be collected with a detailed explanation of variables on questioner for a total of around 3 hours. The data source was the hospital’s information management system (HIMS) registry book (in an emergency, ICU & Burn ward), and patient chart.

### Data processing and analysis

Before data entry, the abstraction checklist is checked for completeness. All checklists were checked for completeness and Cleaned and coded data was entered into SPSS version 25.0. And also analysis of the data was conducted by using SPSS. Both descriptive and analytical statistical procedures were used to summarize the distribution of variables. Descriptive statistics like percentage, mean, and standard deviation were used for the presentation of socio-demographic data and prevalence of burns. Tables were also used for data presentation. The effect of the independent variable on the dependent variable was analyzed by using bivariable and multivariable logistic regression as well as Pearson χ2 test. All explanatory variables with a *p*-value of ≤ 0.2 from the bivariable logistic regression model were fitted into the multivariable logistic regression model to control the possible effect of confounders and finally, the variables which had been independent association with the outcome of the burn patient (death or survive) was identified based on OR, with 95% CI and a *p*-value less than 0.05 were significant. The face and content validity of the tool was examined by the researchers. The Cronbach’s alpha coefficient of the data collection tool was 0.84 for assessing the internal consistency and reliability of the tool. Model goodness-of-fit was checked by Hosmer and Lemeshow test (0.272).

## Result

### Socio-demographic characteristics

**Fig. 2:**
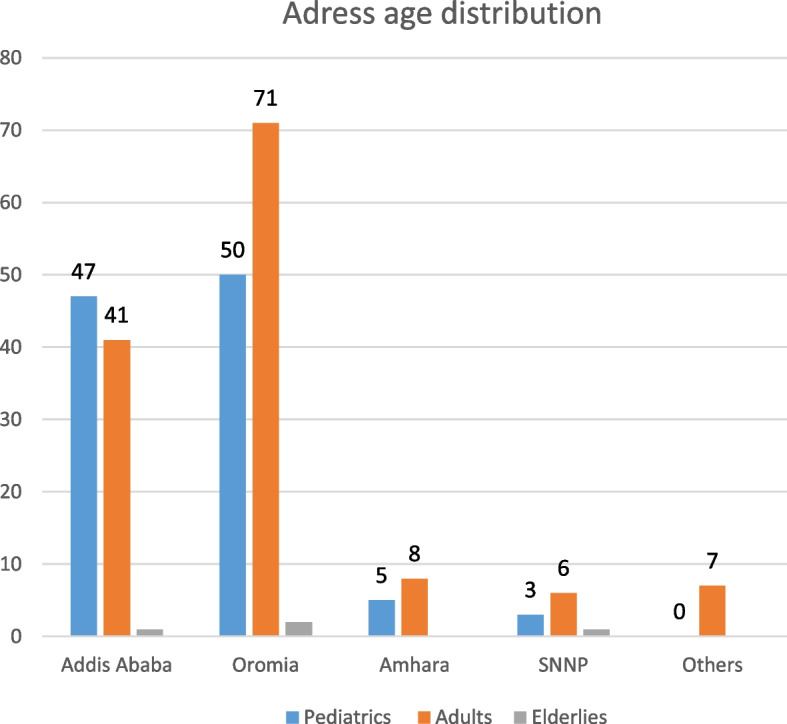
Graphs showing the distribution of address with age distribution among burn patients who visited AaBET hospital from April 1, 2019, to March 31, 2021

**Fig. 3:**
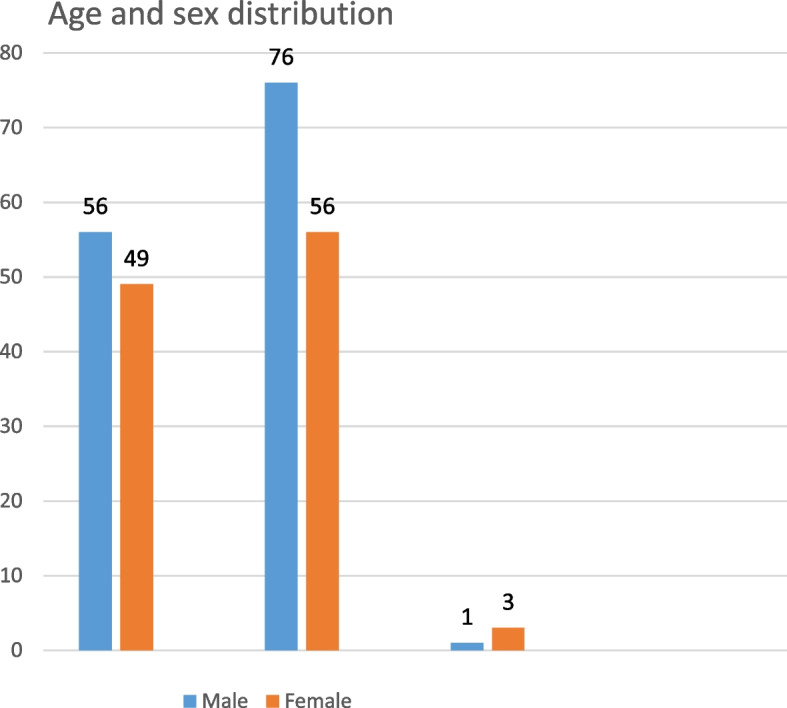
Graphs showing the distribution age and sex distribution among burn patients who visited AaBET hospital from April 1, 2019, to March 31, 2021

A total of 241 patients were eligible for analysis in this study. Among 241 patient’s 132 (55.2%) patients are males and 108(44.8%) were females with a male-to-female ratio of 1.23:1. The age range of the patient was from 5 months to 77 years with a mean age of 18.22(SD -15.02) years. Adults (age 15 to 60 years) are the most affected group 132(54.8%) followed by pediatric age groups (age less than 15 years) (105, 43.6%) and elderly (age greater than 60 years) (4, 1.6%). Except for the elderly group, males predominate females in all age groups (Fig. 3). Most of the patients are from the Oromia region (123, 51%) and Addis Ababa (89, 36.9%) respectively. Others are from the Amhara region (13, 5.4%), Southern Nations, Nationalities, and Peoples' (SNNP) (9, 3.7%), Afar (3, 1.2%), Somalia (1, 0.4%), and Harari (2, 0.8) and Gamebela (1, 0.4%) (Table 1). Except for SNNP males predominate over females in all-region. In Addis Ababa, the Pediatric age group predominates but in other regions, the Adult age is a predominant victim (Figs. 2 and 3).

**Table 1:** Socio-demographic characteristics of burn patients who visited AaBET hospital from April 1, 2019, to March 31, 2021

Category	Frequency	Percentage
Age		
< 15 years	105	43.6%
15-60 years	132	54.8%
> 60 years	4	1.6%
Totals	241	100%
Sex		
Male	133	55.2%
Female	108	44.8%
Totals	241	100%
Regions		
Addis Ababa	89	36.9%
Oromia	123	51%
SNNP	9	3.7%
Amhara	13	5.4%
Others^a^	7	3%
Total	241	100%

^a^Others (Tigray, Hareri, Diredewa, Gamebela, Benshangul, Somalia, Afar)

### Clinical characteristics of the burn patients

#### Triage category and management gave at referring hospital

But, only 54% took documented topical antibiotics application. Other service given from their primary visit area includes tetanus antitoxoid (TAT) administration (15.8 %), Oxygen administration (2.5%), and Compartment release (3.5%) (Table 2).

**Table 2:** Cross-tabulation of referral status of burn patients and referring health facilities among patients who visited AaBET hospital

Category	Referring health care center			
	Public health centers	Private healthcare facility	Public hospital	Total
Referral status				
Serf referral	1	2	1	4
Referred with communication	49	6	67	122
Referred without communication	21	8	34	63
Referral status unknown	6	4	3	13
Total	77	20	105	202

#### Causes, depth, and Extent of burn injury

Scald burn was the major cause (94, 39%) followed by flame burn (81, 33.6%), electrical burn (64, 26.6%), and chemical burn (2, 0.8%). But, Contac burn was not observed in the study. Scald burn was more common in the pediatric age group than in adults by a 3:1 ratio. The electrical burn was caused more in the adult age group with a ratio of adult to pediatric 6.7:1. Flame burns also affected adults more than pediatrics by a 2:1 ratio. Elderly’s burn was caused by flame (3, 75%) and Electrical (1, 25%) burn. Scald and electrical burns were the most common cause of burn-in males while the Flame burn was a major cause of burn-in female patients. Electrical burn is disproportionately encountered in the adult age group and male patients. The chemical burn was observed only in adult age group female patients. Both the sex and age of the patient significantly correlate with the cause of the burn (*p*: < .001). Most burn victims had second-degree superficial (96, 39.8%) followed by Full-thickness (77, 32%), second-degree deep (67, 27.8%), and first-degree burn (1, 0.4%). Pediatric age groups mostly experienced second-degree superficial (56, 53.3%) followed by second-degree deep (30, 28.6%), full-thickness (18, 17.1%), and First-degree burn (1, 1%). Most adults had full-thickness burn (56, 42.4%), followed by second-degree superficial (40, 30.3%) and second-degree deep (36, 27.3%). Elderlies encountered full-thickness (3, 75%) and second-degree deep burn (1, 25%) only. The age of the patient had a significant association with the depth of the burn (*p*<.001). The median TBSA% was 10.5% with a mean of 15.49%, a standard deviation (SD) of 13.78%, and a range from 1% to 64%. Most burn patients had < 10 % (119, 49.4%), followed by 10 to 20% (60, 24.9%), 20 to 30% (31, 12.9%), and > 30% (31, 12.9%). In all age groups pattern is the same as percentages of categorized classes but adult age groups had a higher proportion for a burn with >30% of TBSA% than other age groups. Age has a significant association with the extent of burn (p- .003). Scald burns had a higher proportion of second-degree superficial burns (59, 61.7%) but flame and electrical burns had more full-thickness burns (31, 40.3%) and (40, 51.3%) respectively). The commonest extent of the burn in all types of burn was TBSA% of less than 10% but Flame burns cause more > than 30% of TBSA% burn than scald and electrical in a ratio of almost 2:1. Cause of burn had a significant association with Depth of burn (*p*<.001) as well as Extent of burn injury (*p*=0.003) (Table 3).

**Table 3:** Cause of burn injury and related factors among patients who visited AaBET hospital from April 1, 2019, to March 31, 2021

	Cause of burn injury					*P*-Value
	Scald N (%)	Flame N (%)	Electrical N (%)	Chemical N (%)	Total N (%)	
Overall percentage	94,39%	81,33.6%	64,26.6%	2,0.8%	241,100%	
Age group						
< 15years	71,67.6%	26,24.8%	8, 7.6%	0,0%	105, 43.6%	< .001
15-60 years	23, 17.4%	52, 39.4%	55, 41.7%	2, 1.5%	132, 54.8	
>60years	0,0%	3,75%	1,25%	0, 0%	4, 1.6%	
Sex						
Male	54,40.6%	24, 18%	55,41.4%	0,0%	133,55.2%	<.001
Female	40, 37%	57,52.8%	9,8.3%	2,1.9%	108,44.8%	
The extent of burn injury						
< 10%	48,40.3%	32,26.9%	38,31.9%	1,0.8%	119,49.4%	.003
10-20%	33,55%	17,28.3%	9, 15%	1, 0.5%	60, 24.9%	
20-30%	7, 22.6%	16,51.6%	8,25.8%	0,0%	31, 12.9%	
>30%	6,19.4%	16,51.6%	9,29%	0,0%	31, 12.9%	
Depth of burn injury						
First degree	1,100%	0,0%	0,0%	0,0%	1,0.4%	<.001
Second degree Superficial	59,61.5%	24,25%	12,12.5%	1,1%	96, 39.8%	
Second degree deep	28,41.8%	26,38.8%	12,19.8%	1, 1.5%	67,27.8%	
Full thickness	6, 7.8%	31,40.3%	40,51.9%	0,0%	77, 32%	
The total length of stay in the hospital						
Less than 20 days	61,58.1%	24,22.9%	19,18.1%	1, 0.9%	105,43.6%	.765
20 to 40 days	21,45.7%	12,26.1%	13,28.3%	0,0%	46,19.1%	
40 to 60 days	6,14.3%	20,47.6%	16,38.1%	0,0%	42, 17.4%	
> 60 days	6, 7.5%	25,31.25%	16, 20%	1,1.25%	48, 60%	

#### Anatomic locations of burn injuries

The upper extremity is the most affected body part and is involved in 63.1% of followed by the Lower extremity (56%), anterior trunk (45.6%), face (24.5%), posterior trunk (21.6%), neck (17.8%), Head (12.4%) and genitalia (5%). In 73% of the case, there is the involvement of two or more body parts involvement. 6.2% of patients had associated inhalational injuries.

#### Associated injury and comorbidity

Twelve burn victims (5 %) had associated injuries which 3 people (1.2 %) had blunt chest trauma with hemothorax, 3 people (1.2%) had extremity fractures (1 femoral shaft and 2 clavicular fracture) and 6 (2.5%) had a mild head injury(1 victim had minimal subdural hematoma). 7.5% of all burn victims had comorbidity epilepsy prevalent in 13 patients (5.4%). Other comorbidities include pregnancy (3, 1.2%), hypertension (HTN) (2, 0.8%), psychiatric illness (2, 0.8%), diabetes mellitus (DM), and HIV (human immunodeficiency virus) (1, 0.4% each).

#### Management and outcome at the emergency

About 97.1 % of the patient took analgesia and wound care at the emergency unit. Other care given includes fluid resuscitation for about 46.5%, TAT for 24.9%, endotracheal intubation for 3.3%, wound debridement for 1.7%, fasciotomy for 2.1%, and Iv antibiotics for 21.2% of patients are given in emergency department. One patient require dialysis but was not done because it was not available as well can’t transfer the patient to a dialysis-capable center because the patient was hemodynamically unstable. The mean length of stay of the patient was 39.4 hours with minimum disposal within one hour and a maximum stay of 21 days. Most of the patients were admitted to the ward (206, 85.5%) and ICU (12, 5%). 22 patients (9.1%) were discharged but 1 patient (0.4%) died in an emergency.

#### Management and outcome in ICU and burn ward

Of 13 patients (including one patient from the burn ward) Admitted to ICU 8 was died and 5 transferred to the burn ward with improvement. The mean length of stay in the ICU was 13.05 days (ranging from a minimum of 17 hours to a maximum of 78 days). Among 211 patients admitted to the burn ward: 12 (5.7%) patients continue fluid resuscitation, 78(37%) took IV Antibiotics and all patients took Analgesia and had wound care according to their needs (every other day, once daily, two times per day or three times per day). The mean length of stay for a patient admitted to the burn ward is 39.6 days (range a minimum of 1 day to a maximum of 175 days). 167 (79.1%) patients are discharged without complication, 36(17.1%) patients are discharged with documented complications, 1 patient (0.5%) was admitted to ICU, and 7(3.3%) patients died from the burn unit. Overall, among all burn patients visited during the designed study period, surgical interventions were undergone for 121 (50.2%) patients. Among surgical interventions, a skin graft was done for 106 (44%), wound debridement for 42 (17.4%), tissue flap for 17(7.1%), contracture release for 15(6.2%), and fasciotomy done for 5 (2.1%).

#### Early Complications during a hospital stay

36.1 % of burn victims developed at least one early complication. The top complication was burn wound site infection (30.7%). Others include Burn wound sepsis (22%), hospital-acquired pneumonia (10%), Septic shock (6.2%), catheter-associated urinary tract infection (4.6%), respiratory failure (4.1%), hospital-acquired infection of unknown origin (1.7%), arrhythmia (1.7%), COVID-19 infection (1.7%) and bedsore (1.2%). 26.1% of burn victims developed anemia with a hemoglobin level of less than 10 g/dl. Concerning electrolyte imbalance hyponatremia was the commonest finding among those who had electrolyte determination (37.5 %,), hypokalemia in 18.4%, hyperkalemia in 8.2%, and hypernatremia in 10.1% are also found. Creatinine greater than 1.2 (highest lab value) was found in 6.2% of burn victims.

#### Time-related factors

The earliest time patient presented to the hospital was 30 minutes and the latest time is 120 days with a median of 12 hours. Only 25.7% of burn patients come in the first 24 hours of the period post-burn accident. Length of stay in hospital ranged from 2 hours to 177 days with mean & SD of 37 days and 34.2 days respectively. Length of stay of fewer than 20 days had the highest mortality.

### Factors associated with the outcomes of burn

In General, of 241 burn patients 189(78.4%) were discharged without complications, 36(14.9%) were Discharged with complications and 16(6.6%) died. The commonest long-term complication is the Amputation of the extremity (19, 7.9%) followed by contracture (14, 5.8%) and Hypertrophic Scar of skin graft or wound (3, 1.2%). Of all death, septic shock was identified as the immediate cause of death in 8 patients (50%) followed by multi-organ dysfunction in 4 patients (25%), Respiratory failure in 3 patients (18.8%), and arrhythmia in 1 patient (6.3%). In a bivariate analysis of adult and old age, burn TBSA% of 20 to 30% and greater than 30%, time-lapse 72 hours before arrival to the hospital, early complication while in hospital stay, associated injury, and length of stay less than 20 days are significantly associated with *p*-value <0.2. When the multivariable analysis was done TBSA% of greater than 30% and age greater than 60 were the only variable associated with a *p*-value of < 0.05. TBSA% > 30% increased the mortality rate by 8.7 times and age > 60 years increased mortality by 2.2 times than its counterparts (Table 4).

**Table 4:** Bivariate and multivariate analysis showing association of determinant factor and outcome of burn patient admitted to AaBET hospital from April 1, 2019 to March 31, 2021

	Outcome of burn patients					
	Death N,%	Survival N,%	COR(95%CI)	*P*-value	AOR 95% CI	*P*-value
Age						
< 15 yr.	1(1%)	104(9%)	1.00	.000		
15-60 yr.	14(10.6%)	118(89.4%)	12.34 [1.59-95.45)*	.016		
>60 yr.	1(25%)	3(75%)	34.667[1.726-696.338]*	.012	2.206[1.323-3.69]**	0.037
Sex						
Female	9(8.3)	99(91.7%)	1.00	.000		
Male	7(5.3%)	126(94.7%)	0.611[0.22-1.698]	.345		
Cause						
Chemical	0 (0 %)	2(100%)	1.00	.999		
Scald	0(0%)	94(100%)	1.00	1.000		
Flame	9(11.1%)	72(88.9%)	201934808.8[000…]	.999		
Electrical	7(10.9%)	57(89.1%)	198392092[0000]	.999		
Extent of burn injury						
<10%	2(1.7%)	117(98.3%)	1.00	.000		
10 – 20%	0(0%)	60(100%)	0.00 [000]	.997		
20-30%	3(9.7%)	28(90.3)	6.268[0.999-39.312]*	.050		
>30%	11(35.5%)	20(65.5%)	32.175[6.63-156.2]*	<.001	8.72[1.32-57.33]**	.012
Associated trauma						
Yes	3(23.1%)	10(76.9%)	4.982[1.216-20.249]*	.026		
No	13(5.7%)	215(94.3%)	1.00	.000		
Time-lapse before arrival						
<24 hours	2(3.2%)	60(96.8%)	1.000			
24 to 72 hours	0(0%)	17(100%)	.000	.999		
>72 hour	14(8.6%)	148 (91.4 %)	2.838[0/626-12.867*	.176		
Any early complication						
Yes	15(17.2%)	72(82.8)	31.875[4.13-246.0)*	.001		
No	1(0.6%)	153(99.4%)	1.00	.000		
Any pre-hospital Intervention						
Yes	14(9.8%)	139(90.2%)	4.835 [0.69-37.747]*	.133		
No	1(2%)	48(98%)		.000		
The total length of stay in hospital in days						
Less than 20 days	12(11.4%)	93(88.6%)	6.065[0.765-48.057]*	.088		
20 to 40 days	2(4.3%)	44(95.7%)	2.136(.187-24.398]	.541		
40 to 60 days	1(2.4%)	41(97.6)	1.148[.069-18.913)	.924		
Greater than 60 days	1(2.1%)	47(97.9%)	1.00	.000		

^*^Significant at *p*: < .2 , ** significant at *p*: < 0.05, *COR* Crude odds ratio, *AOR* Adjusted odds ratio, *CI* Confidence interval at 95 %

## Discussion

Our study conducted a review for two consecutive years (from April 1, 2019, to March 31, 2021) on the outcome of burn injury, Most burn victims were Adults whose age range is from 15 to 60 years, which accounts for about 54.7% of the study population. This is comparable to a study done by S.Triphate & S.J.Basenet, Nepal, India which accounts for about 65.4% [46]Predominance of adult age is also described in South Africa and Singapore. The predominance can be explained by the fact that they are the most active groups and are exposed to hazardous environments both at home and at the workplace. In this study males also predominate females with a male-to-female ratio of 1.23:1. The result is consistent with the study of N.A. Forbinake, Cameroon, which consists of 68.3% [47]. The reason may be males are more participants in hazardous workplaces like industries with faulty electrical wires Adults and boys are more active than girls in the pediatric age group. Since the workplace and events during the accident were difficult to access due to the nature of the study and not assessed in this study, we couldn’t conclude specifically. A contemporary, study done by Tesfaye Mulat, Yekatit 12, Addis Ababa, Ethiopia shows female predominance which consists 64.5 % [48]as well by S.Triphate & S.J.Basenet, Nepal which accounts for 55.6 % [46]. This study is explained by the fact that females are predominantly involved in household activities that strongly correlate with fire and hot liquids like cooking and baking. On top of that, they usually handle fire in relatively open spaces [46, 49].

The most frequent cause of injury is scald which accounts for 39% followed by Flame, Electrical and chemical burn (33.6%, 26.8%, and 0.8% respectively). This result was also shown in a study by Tesfaye Mulat, Yekatit 12 hospital but according to S.J.Basenet, Nepal, India Flame was the commonest cause [46, 48, 50]. The variety could be due to population distribution and cultural variation. Scald burn is the number one cause in the pediatric population but adults are affected more by electrical and flame burns. Electrical burn currently shifts epidemiology from previous studies [51]. Usually Male is more affected than a female with a male-to-female ratio of 6.1:1 and adults are more prone than pediatrics with a ratio of 6.7:1. The children’s attitude in playing (stay) at home or in the kitchen with their mother or guardians where drinks and liquid foods are being prepared with inadequate supervision and inability to protect themselves may explain why scald commonest in children. Electrical burn as described earlier, work area exposed male adults are most commonly affected [46, 47, 49, 52]. Moreover, flame burn injuries were the second most common (33.6%) frequent cause of burn injury in this study. It accounts for 75% of elderly’s burns and is the most common cause of burn-in in Female patients with a ratio of females to males of 2.3:1. Asa described earlier working environment will explain it well [6, 53].

The median TBSA% was 10.5% with a mean of 15.49%, SD of 13.78%, and a range from 1- 64%. It is slightly lower than the study done by Tesfaye Mulat, Yekatit 12 Hospital, Addis Ababa, in which the mean was 17.2% [48]. TBSA %< 10 % is the most common accounting almost for half of the cases (49.4%) and higher than the study of Frehiwot Girma, Bahirdar Ethiopia, in which total body surface area was mean of 12.56 and SD OF 8.91% [49, 52]. Higher TBSA% (>20%) is more observed on flame burn than others [49]. This may be due to the nature of the offending agent and the duration of contact with it. A study by Aditya Wardhana, Indonesia shows that the mean TBSA% was 26.7% (range from 0.5% to 100%). This could be due to most patients' sustained flame burn (50%) [54]. The most observed degree of burn is superficial second-degree burn accounting for 39.4%. Pediatric age groups encountered more superficial second-degree burns while adults and the elderly commonly full thickness burns. This could be due to cause of the degree of burn, in which, scald more cause first-degree burn and second-degree superficial while flame and electrical burn cause full thickness & Second degree burn [40, 43, 53, 55].

The upper extremity is the most affected body part and is involved in 63.1% of followed by the Lower extremity (56%), anterior trunk (45.6%), face (24.5%), posterior trunk (21.6%), neck (17.8%), Head (12.4%) and Genitalia (5%). In 73% of the case, there is the involvement of two or more body parts involvement. This is higher but consistent in part involved from a study done in another region of Ethiopia like 43.3% in a study of Frehiwot Girma, Bahirdar, Ethiopia [49] and 45.4% in a study Sielu Alemayehu, Ayder, Mekele, Ethiopia [3]. This could be due to the nature of an offending agent and personal response to the circumstance of events. Twelve burn victims (5 %) had associated injuries in which 3 people (1.2 %) had blunt chest trauma with hemothorax, 3 people (1.2%) had extremity fractures (1 femoral shaft and 2 clavicular fracture) and 6 (2.5%) had a mild head injury(1 victim had minimal subdural hematoma). 7.5% of all burn victims had comorbidity epilepsy prevalent in 13 patients (5.4%). Other comorbidities include pregnancy (3, 1.2%), hypertension (2, 0.8%), psychiatric illness (2, 0.8%), diabetes mellitus, and retroviral infection (1, 0.4% each). Epilepsy is mentioned in various types of research as the commonest comorbidity in 5.6% in a study of S.Triphate & S.J.Basenet, Nepal, 4.1% in a study of Aditya Wardhana, Indonesia, and 7.8 % in frehiwot Girma, Bahirdar, Ethiopia [46, 49, 54]. This could be due risky nature of the disease to end up with a fall at an unsafe place including on causative agents of burn.

Most of the patients come to this hospital by referral system. Among them, 75.6% of patients received some sort of treatment which include, Resuscitation fluid, analgesia, wound care, oxygen administration, fasciotomy, or a combination of them. Gaps are observed in handling the patient, starting available appropriate treatment, and documentation as well as delays in the referral system. This could affect most unstable patients in getting definite management. A similar problem is faced in other countries like Indonesia (according to Aditya Wardhana study), in which, patients wait for a longer time to get a referral to a territory center where the subspecialists are available [54]. This is due to the scarcity of facilities as well as equipment and manpower faced by most developing countries.

In our hospital, all patients had been given necessary routine care but 50% of them needed advanced care including endotracheal intubation and other surgical intervention. According to endotracheal intubation (8, 3.3%), skin graft (106, 44 %), wound debridement (44, 17.1%), tissue flap (17, 7.1%), contracture release (15, 6.2%) and fasciotomy (5. 2.1%) had been done. When comparing to the study of Tesfaye Mulat, Yekatit 12 hospital, Addis Ababa, a surgical operation done here was low. According to this study, 74.7% of patients had delayed splint skin grafts. This may be due to the low sample size (121), the nature of the burn, or improvement in the care of the patient in our hospital [48].

Thirty six point one (36.1%) of burn victims develop at least one early complication while staying in this hospital. The top complication was burn wound site infection (30.7%) and Burn wound sepsis (22%) followed by Anemia of hemoglobin less than 10 g/dl ( 26.1%), hospital-acquired pneumonia HAP (10%), Septic shock (6.2%), CAUTI (4.6%), respiratory failure (4.1%), hospital-acquired infection of unknown origin (1.7%), arrhythmia (1.7%), COVID 19 infection (1.7%) and Bedsore (1.2%). hyponatremia in 37.5 %, Hypokalemia in 18.4%, hyperkalemia in 8.2%, and hypernatremia in 10.1% are the most encountered electrolyte abnormality. 6.2% had a Creatinine of > 1.2g/dl which possibly suggests acute kidney injury. This result is consistent with the study done by N.A. Forbinake in Cameroon [47].

After all, 241 burn patients 189(78.4%) were discharged without complications, 36(14.9%) were Discharged with complications and 16(6.6%) died. The commonest long-term complication is amputation of the extremity (19, 7.9%) followed by contracture (14, 5.8%) and hypertrophic Scar of skin graft or wound (3, 1.2%). The death rate is improved from Tesfaye Mulat study in Yekatit 12, which was 11.6% as well in most African countries like Cameroon (N.A. Forbinake study, 23.4%) [47], Malawi (G. Virich study, 26.4%) [56, 57], Nigeria(A.O. Olandele and J.K Olabanji study, 26%) [58] and some Asian country like Indonesia (Aditya Wardhana study, 24%) [54] and Nepal (S.Triphate & S.J.Basenet study, 25.4%) [46]. Of all death, septic shock was identified as the immediate cause of death in 8 patients (50%) followed by multi-organ dysfunction (MOD) in 4 patients (25%), respiratory failure in 3 patients (18.8%), and arrhythmia in 1 patient (6.3%). There is agreed data from Nepal and Cameroon with the commonest cause of death as septicemia with septic shock followed by MOD, acute respiratory distress syndrome (ARDS) as well acute renal failure, and severe anemia [46, 47].

In General, in this study, of 241 burn patients 189(78.4%) were discharged without complications, 36(14.9%) were discharged with complications and 16(6.6%) died. This is comparable with those conducted in East Africa (7.1%) [59], and Mekele (6.0%) [60]. However, it is higher than studies conducted in the USA at 0.85% [61], Nigeria at 3.8% [39], Egypt at 2.5% [62], Iraq (4.7 %) [63], Dutch (3.2%) [64], Turkey (3.8%) [65], and Pakistan 2.35% [66]. The discrepancy might be explained by the lack of infrastructure, ambulance service, and health facilities in our study setting. This discrepancy might also be due to the lack of burn unit centers in our study setting, and the lack of trained and experienced health professionals dedicated to the care of burn patients compared to the mentioned studies [65]. The finding in this study is lower than a study conducted in Ghana (21.3%) [67], and Tanzania (11.7%) [68]. This discrepancy might be due to the difference in socio-demographic characteristics, and due to the reason that studies in mentioned countries were conducted in tertiary hospitals which are prone to have overflows of burn patients throughout their country and increases the rate of mortality in the hospitals [67, 69, 70]. On the other hand, in our study-setting complex and complicated burn cases are referred to in another area, which might decrease the mortality rate in the study setting.

According to this study in multivariable logistic regression analysis, age > 60 years and TBSA% greater than 30% were significantly associated with burn mortality. The odds of mortality among burn patients were 2.206 times higher among burn patients aged> 60 years than those aged < 15 years [AOR=2.206 (95% CI, 1.323-3.69)]. This study is similar to different studies which showed that the mortality risk was higher in older patients [71, 72]. Natural age advancement impairs wound healing significantly and makes aged burn patients more prone to infections and associated complications and worsens the clinical outcome [73]. Older persons are affected by burns and the resources required to meet their needs may be higher due to their long periods of hospitalization [74].

Regarding the extent of burn injury, the risk of mortality among burn patients was 8.72 times higher in burn patients who had TBSA% greater than 30 than those who had TBSA<10% [AOR=8.72 (95% CI, 1.32-57.33)]. The bigger the burn, the greater the risk of the subsequent burn wound and systemic infections, which are directly associated with increased morbidity and mortality [75, 76]. In this study total body surface area, greater than 30% of burn was times more likely (AOR 8.72; 95% CI 1.32–57.33) to die compared to less than 10% burn area. This study is consistent with the study done in South Gondar zone government hospitals [77], and Bahir Dar, Ethiopia [78]. This is supported by the scientific evidence of those who have a larger body surface area of burn are prone to complex fluid shifts, including system-wide extravasation of fluids into unburned tissues. This results in resultant intravascular hypervolemia which can produce ischemia to body organs, lactic acidosis, and eventual cardiovascular collapse [79, 80], which increases the risk of mortality and complications.

## Strengths and limitations of the study

### Strength

Since there was no published study from AaBET hospital since the start of the hospital, it will give information about the overall burn patient outcome. Furthermore, it will help to identify gaps and give a solution for them.

### Limitations

Since the study was a retrospective documentary review, there were challenges like incomplete documentation at all sites of care( like subsequent day-to-day progress of the patients), an inconsistent subsequent clinical feature of the patient, ineligible handwriting, and no or unclear information for some variables( type of analgesia, amount and dosage of fluid taken at pre-hospital levels). Due to the nature of the study, information gathered by taking history, doing a physical examination, or simple observations are missed (like socioeconomic status, housing condition, circumstance while the event occurs, etc.….)

## Conclusion

The commonest cause of burn was scald burn. Electrical burns and flame burns are more common in adult and elderly age groups. Extremities were by far, the commonest affected body parts. The mortality rate decreased significantly from previous studies. But, disability up on discharge remains high. The extent of burn injury and the age of the patient independently predict the mortality of the patient. Therefore, timely identification and monitoring of burn injury should be necessary to prevent the mortality of burn victims, and it is highly recommended to give priority to preventive measures and improve pre-hospital care service.

## Recommendation

Overall the mortality rate shows a decrement from previous studies as compared to a study done at another site. But burning is still a burden for our countries. To decrease mortality and morbidity, we recommend the following;

### To health professionals

- ➢ I recommend early and timely aggressive resuscitation with an early referral habit where the best care will be given to the patient. Clear documentation at each level of care and possibly teach epileptic patients to avoid unsafe work environments and proper medication use.

### To researchers

- ➢ I also recommend the need for further community-based studies that determine the risk factor, severity of burn injuries, the outcome, and quality of care given at health institutions prospectively with good overall supervision.

### To the Ministry of Health and regional health bureau

- ➢ Time is sensitive for burn victim patients. There should be adequately trained personnel experienced in the management of burn patients. I highly recommend opening well-equipped, both in terms of material and trained personnel, burn units across all regional states. This hopefully will improve the quality of care, and decrease the chance of mortality and disability.

## Data Availability

All data about this study are contained and presented in this document.

## Abbreviations

AaBET: Addis Ababa Burn, Emergency And Trauma Hospital
AOR: Adjusted odds ratio
BMS: Burn Model System
CI: Confidence Interval
COR: Crude Odds Ratio
ED: Emergency department
EMCC: Emergency medicine and critical care
GC: Gregorian calendar
HAI: Hospital-acquired infection
HAP: Hospital-Acquired Pneumonia
HIV: Human Immunodeficiency Virus
HIMS: Health Information Medical Record System
ICU: Intensive care unit
IRB: Institutional Review Board
NDSC: National Data and Statistical Center
NIDRR: National Institute on Disability and Rehabilitation Research
SD: Standard Deviation
SPHMMC: Saint Paul Hospital Millennium Medical College
Spo2: Pulse Oximeter Oxygen Saturation
SPSS: Statistical Package For Social Science
TBSA: Total Body Surface Area
LMICs: Low- And Middle-Income Countries
WHO: World Health Organization
BSc: Bachelor of Science
OR: Odd Ratio
CI: Confidence Interval
TAT: Tetanus Antitoxoid
DM: Diabetes Mellitus
HTN: Hypertension
MOD: Multi-organ dysfunction
